# Interference between overlapping memories is predicted by neural states during learning

**DOI:** 10.1038/s41467-019-13377-x

**Published:** 2019-11-25

**Authors:** Avi J. H. Chanales, Nicole M. Dudukovic, Franziska R. Richter, Brice A. Kuhl

**Affiliations:** 1 Department of Psychology, New York University, 6 Washington Place, New York, NY 10003 USA; 2 Department of Psychology, 1227 University of Oregon, Eugene, OR 97403 USA; 3 Leiden University, Rapenburg 70, 2311 EZ Leiden, Netherlands; 4 Institute of Neuroscience, University of Oregon, Eugene, OR 97403 USA

**Keywords:** Cognitive neuroscience, Neural decoding, Learning and memory, Psychology

## Abstract

One of the primary contributors to forgetting is interference from overlapping memories. Intuitively, this suggests—and prominent theoretical models argue—that memory interference is best avoided by encoding overlapping memories as if they were unrelated. It is therefore surprising that reactivation of older memories during new encoding has been associated with reduced memory interference. Critically, however, prior studies have not directly established why reactivation reduces interference. Here, we first developed a behavioral paradigm that isolates the negative influence that overlapping memories exert during memory retrieval. We then show that reactivating older memories during the encoding of new memories dramatically reduces this interference cost at retrieval. Finally, leveraging multiple fMRI decoding approaches, we show that spontaneous reactivation of older memories during new encoding leads to integration of overlapping memories and, critically, that integration during encoding specifically reduces interference between overlapping, and otherwise competing, memories during retrieval.

## Abstract (web summary)

Interference from overlapping memories can cause forgetting. Here, the authors show using fMRI decoding approaches that spontaneous reactivation of older memories during new encoding leads to integration, and less interference, between overlapping items.

## Introduction

Memory failure, or the inability to bring a target memory to mind, is as ubiquitous as it is frustrating. One of the primary causes of memory failures is interference from overlapping memories. Namely, when multiple memories share features, retrieving any one of those memories is more difficult, relative to an “interference-free” situation where memories do not overlap. Memory interference can be conceptualized as competition that occurs during acts of retrieval^1,2^, with the activation of non-target memories negatively influencing successful retrieval of target memories. However, an important and intriguing idea is that interference that occurs at retrieval is partly—if not largely—determined by how memories are encoded. Behavioral and neuroimaging studies have supported this idea by demonstrating that encoding-related factors can influence expressions of interference at retrieval^3–10^. There are, however, mechanistically distinct ways in which factors during encoding may influence interference at retrieval.

Intuitively, it would seem that the best way to avoid memory interference is to keep representations of overlapping memories as distinct as possible—indeed, this is a primary focus of theoretical accounts of how the hippocampus resolves interference^11,12^. It is therefore somewhat surprising that reactivation of overlapping memories during new encoding has been associated with reduced interference^8,9^. One potential account of this relationship is that reactivation allows for older memories to be integrated with newer memories^13–15^. By this account, links are formed between overlapping memories, resulting in relationships that are cooperative instead of competitive^16^. Consistent with this perspective, several neuroimaging studies have found that reactivation of overlapping memories during new learning predicts better performance on tests requiring integration^17–20^. Moreover, behavioral studies have found that integration strategies during learning can powerfully reduce memory interference^3–7,16^. Together, these findings motivate an account wherein reactivation during new learning promotes integration, which in turn reduces interference during retrieval. While this proposed relationship between reactivation, integration, and interference is motivated by prior findings, it has not been directly established. Moreover, it is also possible that reactivation reduces interference via an entirely distinct mechanism: by triggering the differentiation of overlapping memories^21–23^. Thus, in order to understand how reactivation of overlapping memories during encoding reduces interference, it is essential to understand the specific computations performed upon—or triggered by—reactivated memories.

Here, we report a novel behavioral paradigm in which we experimentally manipulate activation of overlapping (non-target) memories during target memory retrieval. We first establish that activating overlapping memories during retrieval produces a behavioral interference cost. Next, we show that reactivating overlapping memories during encoding powerfully reduces this interference cost during retrieval. Finally, leveraging this behavioral paradigm and fMRI multivoxel pattern analyses, we test a mechanistic account of how reactivation during encoding reduces interference at retrieval. Namely, by teasing apart measures of reactivation and integration during the encoding of new associations, we find that reactivation and integration are related to each other, but that integration is a more direct predictor of interference during memory retrieval. These findings support the conclusion that reactivation creates an opportunity for older and newer memories to be integrated, which in turn predicts the degree to which interference between overlapping memories is experienced during retrieval.

## Results

### Activating overlapping memories interferes with retrieval

**Fig. 1:**
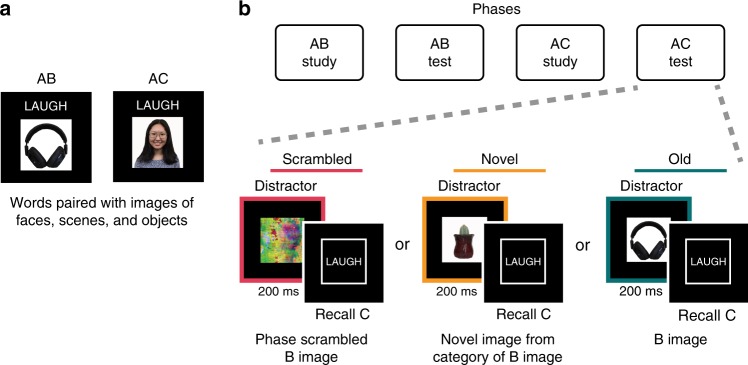
Experimental design. **a** Across all experiments, subjects studied sets of overlapping word-image pairs (AB, AC pairs). Overlap was created by pairing two images (B and C terms) with the same word (A term). **b** Across all experiments, all AB pairs were studied (AB Study phase) and tested (AB Test phase) before AC pairs were studied/tested (AC Study phase/AC Test phase). The AC Test phase contained a critical manipulation in which each test trial was preceded by a briefly presented distractor image (200 ms). For Experiments 1–4, the distractor image was either a phase-scrambled version of the B image (Scrambled condition), a novel image from the same visual category as the B image (Novel condition), or the original B image (Old condition). For the behavioral pilot of the fMRI experiment and for the fMRI experiment, the Scrambled condition was eliminated.

While it is often assumed that memory interference is attributable to activation of overlapping memories during retrieval^1,24,25^, our first aim was to develop a behavioral paradigm in which we manipulated activation of overlapping memories during retrieval and to specifically measure the corresponding behavioral cost. Experiments 1 and 2 used the same general paradigm and are described together. Both experiments began with an initial study phase (AB Study) during which participants learned word (A)–image (B) pairs (Fig. 1a). This was followed by a test phase (AB Test). After all AB pairs were studied and tested, participants completed a second study phase (AC Study) in which all of the previously studied words (A) were paired with new images (C). This created pairs of overlapping memories (i.e., AB and AC pairs contained an overlapping element, A). Participants were then tested on the new AC pairs (AC Test). This design was modeled after classic memory interference paradigms^6^. However, the novel and critical manipulation in our experiments is that all AC test trials were preceded by a briefly presented (200 ms) distractor image. This distractor either consisted of the original B image (Old condition), a novel image from the same visual category as the B image (Novel condition) or a phase-scrambled version of the B image (Scrambled condition; Fig. 1b).

Although all AC Test trials contained a distractor, we predicted that the degree of interference would vary across conditions. Because the novel images represented a salient and high-level distractor, we anticipated that the novel images would be more disruptive than the scrambled images. Our critical prediction, however, was that the Old condition would yield even greater interference than the Novel condition. This prediction was based on the idea that Old images would activate a competing association (AB), whereas Novel images, while irrelevant, would not activate a competing association. However, the opposite prediction that Novel images would be more disruptive than Old images is a reasonable alternative in that Novel images might be expected to more strongly capture attention than repeated (Old) images. The only difference between Experiments 1 and 2 was in how memories were tested. In Experiment 1, during the AB/AC tests, subjects recalled the specific name of each image aloud, whereas in Experiment 2 subjects responded via button press to indicate the visual category (face, object, scene) of each recalled image. The rationale for using different testing procedures across experiments was to generalize any interference effects across item-specific verbal recall (Experiment 1) and button-press measures (Experiment 2). While verbal recall allows memory to be measured more precisely, button-press measures are much easier to collect during fMRI scanning.

**Fig. 2:**
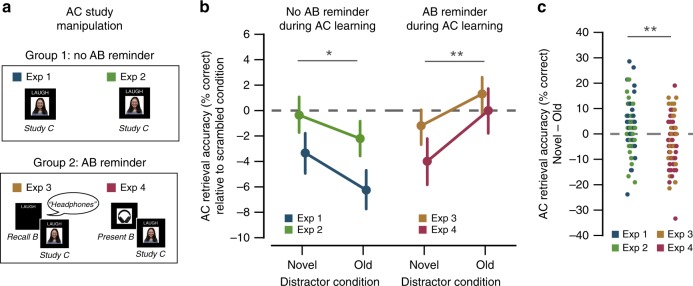
Encoding factors influence interference during retrieval. **a** Illustration of the AC Study manipulations across Experiments 1–4. In Experiments 1 and 2, AC pairs were studied without explicit reminders of AB associations. In Experiment 3, subjects retrieved each AB association immediately prior to studying overlapping AC associations. In Experiment 4, each B image was briefly presented (200 ms) immediately prior to the presentation of each overlapping AC Study trial. **b** AC retrieval accuracy across Experiments 1–4. For standardization across experiments, the Scrambled condition is used as a baseline, with accuracy in the Novel and Old conditions shown relative to this baseline (see Supplementary Table 2 for all accuracy values). When there were no explicit reminders of AB associations during AC Study (Experiments 1 and 2; left columns), AC retrieval accuracy was significantly worse in the Old condition (B image as distractor during AC Test) than the Novel condition (novel image as distractor during AC Test). However, when there were explicit reminders of the B image during AC Study (Experiments 3 and 4; right columns), this pattern was reversed: AC retrieval accuracy was higher in the Old condition than the Novel condition. Thus, reactivating B images during AC Study significantly attenuated interference between AB and AC associations during retrieval. **c** Distribution of the difference scores in AC retrieval accuracy for the Novel vs. Old conditions for Experiments 1–4. Individual dots represent the difference scores (Novel–Old) for individual subjects. Notes: Error bars reflect s.e.m.; **p < 0.01, *p < 0.05 by ANOVA.

Accuracy for the AB Tests is reported in Supplementary Table 1. Of critical interest, however, was accuracy for the AC tests as a function of the distractor type (Fig. 2; also see Supplementary Table 2 for full report of accuracy by condition). An analysis of variance (ANOVA) with factors of Experiment (1, 2) and distractor type (Old, Novel, Scrambled) revealed a significant main effect of distractor type (*F*_2,156_ = 7.64, *p* *<* 0.001) and no interaction between distractor type and Experiment (*F*_2,156_ = 1.87, *p* *=* 0.16). Follow-up comparisons revealed marginally lower accuracy in the novel compared to the scrambled condition (*F*_1,78_ = 2.86, *p* *=* 0.095) with no interaction by experiment (*F*_1,78_ = 1.86, *p* *=* 0.18). Most critically, accuracy was significantly lower in the Old condition compared to the Novel condition (*F*_1,78_ = 4.56, *p* *=* 0.036), with no interaction by experiment (*F*_1,78_ = 0.23, *p* *=* 0.63). Accuracy in the Old condition was also significantly lower than accuracy in the scrambled condition (*F*_1,78_ = 16.38, *p* *<* 0.001), with a marginal interaction between condition and experiment (*F*_1,78_ = 3.76, *p* *=* 0.056). Reaction time (RT) data were only collected for Experiment 2, but these data complemented the accuracy data (Supplementary Table 3). Considering all three conditions (Old, Novel, Scrambled) and only including RTs on correct trials, there was a highly significant main effect of distractor type (*F*_2,78_ = 28.1, *p* *<* 0.001). Follow-up *t* tests revealed that participants were slower to respond on novel trials than scrambled trials (*t*_39_ = 4.50, *p* *=* 0.001) and, most critically, slower on Old trials than Novel trials (*t*_39_ = 2.69, *p* *=* 0.01).

Collectively, data from Experiments 1 and 2 clearly establish that activating an overlapping memory at the time of retrieval produced an interference cost that exceeded any interference due to low- or high-level visual distraction. Importantly, because this paradigm involved directly manipulating the activation of overlapping memories during memory retrieval and measuring the corresponding interference cost, it is ideally suited for our critical question of establishing how encoding-related factors influence competitive dynamics between overlapping memories during retrieval.

### Encoding-related factors influence interference at retrieval

Having demonstrated that activating an overlapping memory interferes with retrieval of a target memory, we next tested whether encoding-related factors influence this interference cost. Specifically, we tested whether activating overlapping memories during encoding reduces the cost of activating overlapping memories during retrieval. The means by which we activated overlapping memories during encoding differed slightly across Experiments 3 and 4. In Experiment 3, participants were instructed to retrieve the original B image immediately prior to encoding the overlapping AC pair, whereas in Experiment 4, the B image was briefly presented (200 ms) immediately prior to studying the overlapping AC pair. In Experiments 3 and 4, recall was tested by verbal report (identical to Experiment 1). To be clear, Experiments 3 and 4 were not intended to isolate a specific mechanism at encoding that reduces interference during retrieval. Rather, these experiments were motivated by prior evidence that reactivation of overlapping memories during encoding is associated with reduced interference-related forgetting^8,9^. Thus, we sought to conceptually replicate this finding before turning to an fMRI study that would dissociate the contributions of reactivation and integration.

Accuracy for the AB and AC Tests is reported in Supplementary Tables 1 and 2, respectively. It should be noted that AC recall accuracy in the Scrambled condition (the baseline condition) was lower in Experiments 3 and 4 than in Experiment 1 (Experiment 1 vs. 3: *t*_78_ = 10.49, *p* *<* 0.001; Experiment 1 vs. 4: *t*_78_ = 6.28, *p* *<* 0.001), indicating that retrieving (Experiment 3) or re-presenting (Experiment 4) the B image during AC learning carried some cost to subsequent memory for the AC association. Again, however, of critical interest was AC recall accuracy as a function of distractor condition (Fig. 2; also see Supplementary Table 2). Of particular interest was whether activating AB associations during AC Study would specifically reduce the cost of activating these same associations during AC Test (i.e., the Old condition). An ANOVA with factors of Experiment (3, 4) and distractor type (Old, Novel, Scrambled) revealed a significant main effect of condition (*F*_2,156_ = 4.64, *p* *=* 0.01), with no interaction by Experiment (*F*_2,156_ = 0.77, *p* *=* 0.46). However, the pattern of results was markedly different compared to Experiments 1 and 2. While presenting the Novel image reduced recall accuracy relative to presenting the Scrambled image (*F*_1,78_ = 5.26, *p* *=* 0.03) (as in Experiments 1 and 2), presenting the Old image was no more disruptive than presenting a Scrambled image (*F*_1,78_ = 0.33, *p* *=* 0.57). Strikingly, recall accuracy in the Old condition was now significantly higher than accuracy in the Novel condition (*F*_1,78_ = 8.62, *p* *=* 0.004), fully reversing the pattern from Experiments 1 and 2. [Note: none of these comparisons interacted with Experiment (*p*s > 0.2).] Thus, (re)activating overlapping memories during encoding dramatically influenced the cost associated with activating these same memories during retrieval. This was confirmed by a highly significant interaction of experiment group (1/2 vs. 3/4) and distractor type (old vs. novel) (*F*_1,158_ = 12.94, *p* *<* 0.001; Fig. 2c).

### fMRI measures of encoding states

The results from Experiments 1 and 2 demonstrate that a brief reminder of an overlapping memory can disrupt retrieval of a target memory, establishing an interference effect that specifically occurs during memory retrieval. Experiments 3 and 4 establish the critical point that this interference effect is highly dependent on the manner in which memories were encoded. Namely, (re)activating overlapping (old) associations during the encoding of new associations markedly reduced interference between these memories during retrieval. While one potential interpretation of the results of Experiments 3 and 4 is that (re)activating the old associations during new learning resulted in integration of old and new associations (thereby reducing interference), there are, as we describe above, other mechanistically distinct ways in which reactivation of overlapping associations during new learning might reduce interference. Thus, our final aim was to tease apart the degree to which reactivation and integration occur during new learning and to assess how/whether each of these phenomena relate to interference during retrieval.

**Fig. 3:**
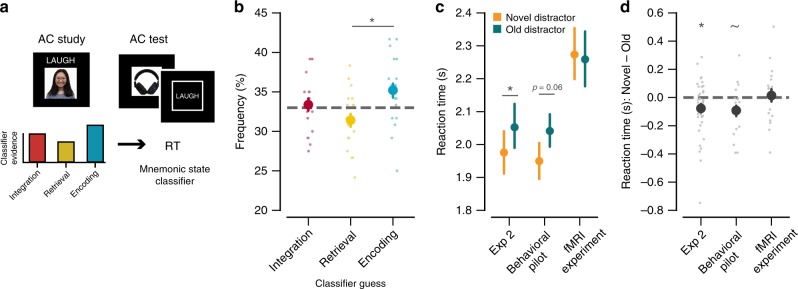
fMRI study. **a** Data from an independent fMRI study^20^ were used to train a pattern classifier to discriminate between three mnemonic states: encoding, retrieval, and integration. This classifier was then applied to (tested on) each AC Study trial. Mnemonic state evidence derived from the classifier during AC Study trials (depicted by the red, yellow, and blue bars) was then used to predict interference, as reflected in reaction times, during AC Test trials. Each dot represents data from an individual subject. **b** For each AC Study trial, the mnemonic state with the strongest classifier evidence represented the “classifier guess” for that trial. Consistent with the fact that subjects’ instructed goal was to encode the new AC associations, the most frequent classifier guess was for an “encoding state.” **c** Reaction time data for AC Test trials in Experiment 2 (left), the behavioral pilot for the fMRI experiment (center), and the fMRI experiment (right). **d** Reaction time difference scores (Novel–Old) for AC Test trials in Experiment 2 (left), the behavioral pilot for the fMRI experiment (center), and the fMRI experiment (right). Each dot represents a difference score for an individual subject. Notes: Error bars reflect s.e.m.; *p < 0.05, ~*p* < 0.1 by *t* test.

To tease apart reactivation and integration during new learning, we conducted an fMRI study and used decoding methods to separately index reactivation and memory integration processes during the encoding of overlapping memories. A key benefit of using fMRI in this capacity is that it allowed us to covertly measure spontaneous reactivation and integration. Of critical interest was whether reactivation and/or integration processes at encoding predicted the degree of interference on corresponding retrieval trials (Fig. 3a). Toward this end, we used a paradigm similar to Experiment 2, which relied on button-press measures, as opposed to verbal reports, to test AC memory. In particular, we designed the experiment to focus on interference effects as reflected in RTs. The rationale for using button-press measures was that they are easier to record during fMRI scanning. The rationale for focusing on RT-based interference effects (as opposed to accuracy-based interference effects) was that accuracy-based analyses often suffer from low power due to small bin sizes (e.g., if subjects are highly accurate, there will be few “forgotten” trials), whereas RT-based analyses can exploit variance across “remembered” trials (as detailed below). We made only a few modifications relative to Experiment 2. These modifications were: (1) we eliminated the Scrambled condition and only included the Novel and Old conditions in order to increase statistical power and (2) in an effort to increase the likelihood that participants reactivated Old memories during new learning, we added an AB Exposure phase to strengthen the original AB memories (see Methods). Importantly, it was anticipated—based on results from Experiments 3 and 4—that increased reactivation of Old memories during new learning might reduce overall interference costs in the Old condition (potentially by promoting integration). However, the goal of the fMRI experiment—following the logic from our prior work^20^—was to relate trial-by-trial variability in classifier measures to behavioral performance. As described above in Experiment 2, the cost of activating overlapping memories during retrieval was robustly reflected in RTs (i.e., slower RTs for the Old vs. Novel conditions), validating the use of RTs to index interference costs (Fig. 3c). As a further validation, we ran a new behavioral pilot that matched the paradigm for the fMRI study almost exactly (see Methods). For this behavioral pilot, RTs were marginally slower for the Old vs. Novel trials (*t*_20_ = 1.97, *p* *=* 0.063), qualitatively identical to the RT results from Experiment 2 (Fig. 3c). Combining data across Experiment 2 and the behavioral pilot, the RT difference for Old vs. Novel trials was highly significant (*F*_1,59_ = 9.45, *p* *=* 0.003) and did not interact with Experiment (*F*_1,59_ = 1.04, *p* *=* 0.31). For the actual fMRI experiment, however, we did not observe a significant difference in RTs between Old and Novel trials (*t*_19_ = 0.59, *p* *=* 0.72; Fig. 3c), but it is important to note that RTs were overall much slower in the scanner than during the behavioral pilot (*t*_39_ = 2.91, *p* *=* 0.006). Moreover, as described above (also see Methods) both the fMRI Pilot study and the fMRI experiment included an extra repetition of each AB association, which was specifically intended to increase reactivation of old memories during AC learning. It was expected (based on results from Experiments 3 and 4) that increased reactivation might result in reduced interference costs. However, the critical aim of the fMRI study was to predict trial-by-trial variability in RTs from fMRI-derived measures of integration and/or reactivation.

### Integration during encoding predicts subsequent interference

To estimate the degree of integration on each AC Study trial, we used multivoxel pattern classification analyses. Specifically—and as detailed in the Methods—we trained a pattern classifier to discriminate between three different “memory states” (encoding, retrieval, and integration) using data from an entirely independent fMRI study previously reported by Richter et al.^20^. In the study by Richter et al.^20^, subjects studied AB and AC associations (similar to the current study), but subjects were explicitly instructed during AC Study trials to alternately engage in each of the memory states (encoding, retrieval, integration). This prior study established that an “integration state” can be successfully decoded from fMRI activity patterns using across-subject classification and, critically, that classifier evidence for an integration state during learning predicts subsequent performance on a behavioral test of AB/AC integration.

To apply the data from Richter et al.^20^ to the present study, we concatenated data across all subjects from Richter et al.^20^ and trained a single pattern classifier to discriminate between the three memory states. This trained classifier was then applied to (tested on) each trial from each subject in the present study. Because subjects in the current study were never instructed to integrate the AB and AC associations, there was, of course, no way to index classification accuracy in the present study. Rather, for each trial the classifier indexed the amount of evidence for each memory state and the state with the highest evidence on that trial constituted the classifier’s “guess.”

First, to generally characterize the performance of the classifier, we computed the frequency of guesses for each mnemonic state (Fig. 3b). Since the instruction given to participants in the current study was simply to learn the new AC pairs, we anticipated that the classifier would guess “encode” most frequently. Indeed, the classifier guessed encode more frequently than retrieve (*t*_19_ = 2.41, *p* *=* 0.026). The frequency of integrate guesses was numerical between encode and retrieve guesses, with no significant differences between the frequency of integrate vs. encode guesses (*t*_19_ = 1.17, *p* *=* 0.25) or integrate vs. retrieve guesses (*t*_19_ = 1.51, *p* *=* 0.15).

Next, we turned to our main question of whether variability in integration during AC Study trials predicted subsequent interference between overlapping memories. For this analysis, integration strength on each trial was indexed by classifier evidence for an integration state. We hypothesized that stronger classifier evidence for integration during AC Study should predict less interference from AB associations during AC retrieval. In other words, we predicted that integrated AB–AC associations were less likely to suffer from interference. Critically, based on the idea that integration specifically reduces interference between overlapping memories, we predicted that integration would benefit retrieval when the distractor was an overlapping memory (Old condition), but not when the distractor was a completely unrelated image (Novel condition).

**Fig. 4:**
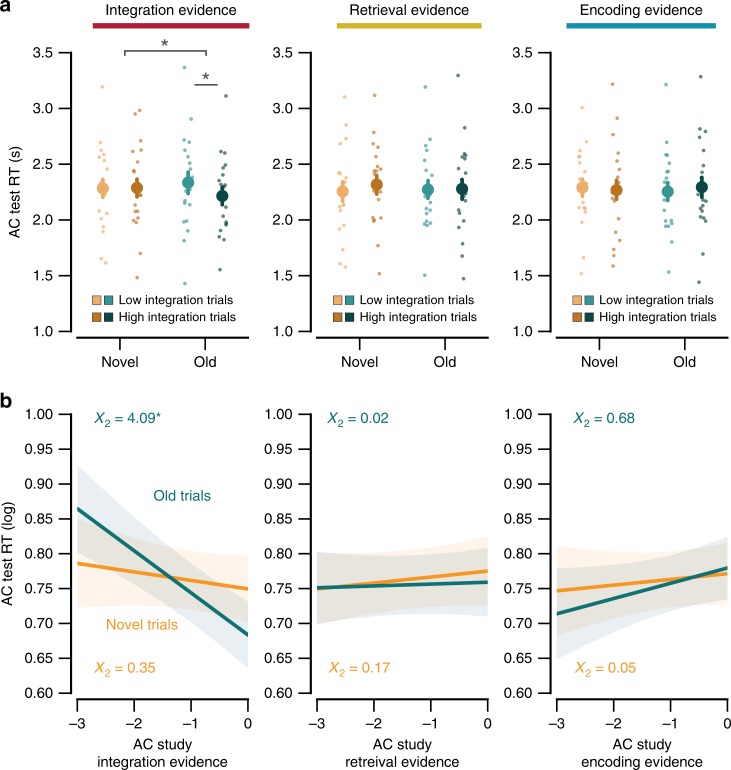
Integration during learning predicts resistance to interference. **a** Reaction times (RTs) during AC Test as a function of evidence from the memory state classifier during AC Study. Novel and Old AC Study trials were median split based on classifier evidence (high vs. low) for integration (left column), retrieval (middle column), and encoding (right column). There was a selective relationship between integration evidence and RTs in the Old condition (*p* = 0.031), reflecting less interference (faster RTs during AC Test) for high compared to low integration during AC Study. Thus, integration specifically protected against interference that occurred when overlapping (Old) associations were active. The interaction between condition (Novel, Old) and integration evidence (high, low) was also significant (*p* = 0.046). Each dot represents data from an individual subject. **b** As a complementary analysis, mixed-effects models were used to predict RTs during AC Test from classifier evidence (as a continuous variable) during AC Study. Left column: for the Old condition, integration evidence during AC Study trials again predicted less interference (faster RTs) during AC Test (*p* = 0.043). However, for the Novel condition, integration evidence during AC Study trials did not predict reaction times during AC Test trials. Neither retrieval evidence (middle column) nor encoding evidence (right column) predicted reaction times during AC Test trials in either the Old or Novel conditions. Notes: Error bars reflect s.e.m; shaded regions reflect 68% confidence interval; *p < 0.05 by ANOVA, *t* test or *χ*^2^ test.

To test for a relationship between integration and interference, we median split all AC Study trials according to the strength of integration evidence (high vs. low) and then obtained corresponding RTs from the AC Test trials. Median splits were separately performed for each subject and each condition (Old, Novel). Within each condition, separate median splits were performed for each visual category group (e.g., B = face, C = scene) and results were then averaged across the different visual category groups; this controlled for any potential confounds due to visual category group. For the Old condition, RTs during AC Test trials were significantly faster when integration evidence during AC Study was high compared to when it was low (*t*_19_ = 2.34, *p* *=* 0.031; Fig. 4a). In other words, high integration during AC Study was associated with lower interference if the old image was presented again during AC retrieval. Critically, integration evidence during AC Study was unrelated to RTs during AC Test trials in the Novel condition (*t*_19_ = 0.12, *p* *=* 0.91; Fig. 4a). Moreover, there was a significant interaction between integration evidence during AC Study trials (high, low) and condition (Old, Novel), indicating that integration evidence was a stronger predictor of reduced interference costs in the Old condition than in the Novel condition (*F*_1,19_ = 4.55, *p* *=* 0.046). Interestingly, “high integration” trials in the Old condition tended to exhibit faster RTs (*M* *=* 2216 ms) than high integration trials in the Novel condition (*M* *=* 2288 ms; *t*_19_ = 1.80, *p* *=* 0.088). While only a trend, this result is qualitatively similar to our findings from Experiments 3 and 4, which showed that if encoding conditions promote reactivation (and potentially integration), activating B memories during AC Test (i.e., the Old condition) can actually be beneficial, relative to a condition where the distractor is unrelated to the C item (i.e., the Novel condition).

As a complementary analysis, we again tested for a relationship between integration and interference, this time using mixed-effects linear regression models. The models tested whether integration evidence on individual study trials predicted subsequent RTs during corresponding test trials. Here, integration evidence was treated as a continuous measure, as opposed to a categorical variable (high vs. low in the prior analysis). Separate models were run for Old and Novel Test trials. Importantly, we controlled for potential effects of visual category information by including the B/C image category pairings (e.g., face/scene, face/object, etc.) as a random-effect term in each model. For Old trials, there was a significant effect of integration strength on RTs during test (*X*_2_ = 4.09, *p* *=* 0.043; Fig. 4b, left column). Specifically, higher levels of integration evidence were associated with faster RTs (reduced interference) during retrieval (*β* = −0.057; SE = 0.027). For Novel trials, there was no effect of integration strength on RTs during test (*X*_2_ = 0.35, *p* *=* 0.55; Fig. 4b, left column). Furthermore, neither retrieval evidence nor encoding evidence predicted RTs on either the Old trials (retrieval: *X*_2_ = 0.02, *p* *=* 0.89; encoding: *X*_2_ = 0.68, *p* = 0.41; Fig. 4b, middle and right columns) or on the Novel trials (retrieval: *X*_2_ = 0.17, *p* = 0.68; encoding: *X*_2_ = 0.05, *p* = 0.82; Fig. 4b, middle and right columns). Thus, consistent with predictions, integration evidence during encoding specifically benefited memory retrieval when overlapping memories were active.

### Reactivation vs. integration

**Fig. 5:**
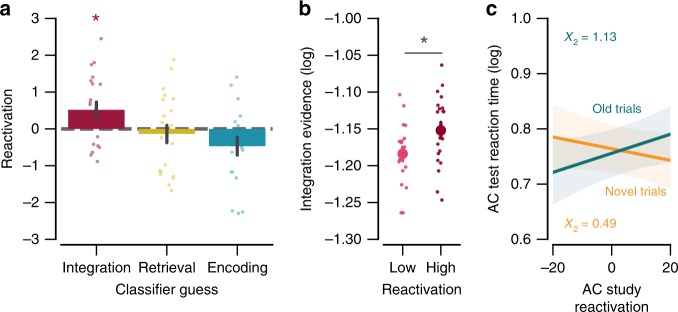
Relationship between reactivation and integration. **a** Significant reactivation of old memories was observed only on trials for which the mnemonic state classifier guessed “integration” (*p* = 0.025). Each dot represents data from an individual subject. **b** High reactivation trials were associated with greater integration evidence compared to low reactivation trials (*p* = 0.048). Each dot represents data from an individual subject. **c** There were no significant relationships between reactivation during AC Study and reaction times during AC Test for either the Old or Novel conditions. Shaded regions reflect 68% confidence interval. Notes: Error bars reflect s.e.m.; *p < 0.05 by *t* test.

Having established that integration protected against interference from overlapping memories, we next sought to measure reactivation of overlapping memories during encoding and to test whether reactivation was related to integration and/or predicted interference between overlapping memories. To measure reactivation, we trained subject-specific pattern classifiers to discriminate between the three visual categories of images (faces, scenes, objects) using data from a Visual Category Localizer (see Methods). The classifier was trained on data from ventral temporal cortex (VTC), motivated by prior evidence of robust visual category reactivation in this area^18,26–28^, and, more specifically, by prior evidence relating reactivation in VTC to measures of integration^20^. The trained classifier was then tested on each trial from the AC Study phase. A reactivation score was computed for each Study Trial, indexing the level of evidence for the Old (reactivated) image (see Methods). To test for a relationship between reactivation and integration, we first binned each subjects’ Study Trials according to the mnemonic state “guessed” by the state classifier (integration, retrieval, encoding). Reactivation scores were then averaged across all trials within each of these bins. Reactivation strength significantly varied across these three bins (*F*_2,38_ = 4.10, *p* = 0.025; Fig. 5a). Interestingly, reactivation was significantly above chance during trials that were labeled as integration (*t*_19_ = 2.37, *p* = 0.028), but not during trials labeled as retrieve (*t*_19_ = −0.55, *p* = 0.586) or encode (*t*_19_ = −1.99, *p* = 0.061). As a complementary analysis, we also binned trials according to reactivation strength (high vs. low, as defined by median split) and then compared continuous measures of integration evidence for these two bins. “High” reactivation trials were associated with significantly greater integration evidence than “low” reactivation trials (*t*_19_ = 2.11, *p* = 0.048; Fig. 5b), again confirming a positive relationship between reactivation and integration.

Having established a relationship between reactivation and integration, we next tested whether reactivation alone predicted interference during AC test trials. To do so, we ran linear mixed-effects models using the reactivation strength on individual trials to predict the RTs during subsequent test trials (Fig. 5c). Reactivation strength was not associated with faster RTs on Old trials (*X*_2_ = 1.13, *p* = 0.29) or Novel trials (*X*_2_ = 0.49, *p* = 0.48). We next tested whether integration was a better predictor of subsequent interference than reactivation. To do so, we compared a linear mixed-effects model that included both trial-level reactivation and integration scores during study as predictors of subsequent RTs (on Old test trials) to separate models that excluded either integration or reactivation measures. While adding integration as a predictor to a model with just reactivation significantly improved the model’s performance (*X*_2_ = 4.08, *p* = 0.043), adding reactivation as a predictor to a model with just integration did not improve the model’s performance (*X*_2_ = 1.26, *p* = 0.26). Put another way, integration evidence predicted subsequent RTs above and beyond what was accounted for by reactivation strength. These results are consistent with our prediction that reactivation should be related to integration^20^ and that integration is a specific mechanism by which reactivation can reduce interference.

## Discussion

Here, across a series of behavioral and neuroimaging studies, we provide a specific, mechanistic account of how integration and reactivation during learning influence interference between overlapping memories during retrieval. Our initial studies (Experiments 1 and 2) establish a critical behavioral measure reflecting the cost of activating an overlapping memory during retrieval. Importantly, these studies demonstrate that activating an overlapping memory carries a cost above and beyond the cost associated with visual distraction (i.e., novel images or visual noise). Having established this specific interference cost, we next demonstrated (Experiments 3 and 4) that factors at encoding can powerfully reduce interference from overlapping memories at retrieval. Namely, activating overlapping memories during encoding reduces interference if these overlapping memories are activated again at retrieval. However, Experiments 3 and 4, on their own, do not establish why activating overlapping memories during encoding reduces interference during retrieval. To address this ambiguity, we conducted an fMRI study in which we teased apart spontaneous reactivation of overlapping memories during encoding from neural evidence of memory integration. We show that while reactivation of overlapping memories during encoding was positively correlated with memory integration, interference from overlapping memories during retrieval was more directly predicted by integration evidence than by reactivation. Collectively, these findings provide compelling and mechanistically specific evidence for a relationship between encoding-related factors and interference during memory retrieval.

Although our use of an AB–AC paradigm to measure memory interference is in keeping with a long history of behavioral memory studies, our paradigm differs from typical AB–AC paradigms in a key way: it was specifically designed to isolate the negative influence that overlapping associations (AB) exert during the retrieval of a target association (AC). That is, rather than focusing on overall AC retrieval accuracy, as is typical in AB–AC paradigms^6,8,9^, we focused on the cost associated with activating an overlapping association during retrieval, comparing this cost against control conditions (a novel image or visual noise). Put another way, the critical design feature of our behavioral paradigm is that we manipulated activation of overlapping memories during retrieval^29,30^. While this is a subtle methodological point, it is essential for teasing apart different accounts for how and why encoding-related factors might reduce interference. Of particular importance in the present study was to rule out the possibility that integration during encoding might yield an overall (non-selective) benefit in encoding strength^31^. That is, if we had not manipulated activation of AB associations during AC retrieval, then for any observed relationship between integration evidence and AC retrieval performance, it would be impossible to determine whether integration specifically reduced interference among overlapping associations or whether it simply promoted stronger encoding. Thus, the fact that integration evidence in the present study predicted retrieval performance in the interference condition (the Old condition)—and not in the control condition (the Novel condition)—provides critical evidence that integration during encoding protected against interference during retrieval.

One of the unique aspects of our fMRI approach is that we indexed memory integration by using pattern classifiers that were trained on data from an entirely independent sample of subjects^20^. In the task used for the training data, subjects were explicitly instructed (on some of the trials) to integrate across AB and AC associations. Importantly, we have previously shown, with this independent data set, that classifier-derived evidence for memory integration during learning predicts the ability to link across associations during retrieval^20^. In other words, this training data has previously been validated as being predictive of behavioral expressions of memory integration. One of the benefits of using this across-study classification approach is that it allowed us to covertly measure spontaneous integration. In fact, subjects in the current study had no reason to suspect that the experiment had anything to do with memory integration. Thus, we were able to measure the effects of memory integration, on a trial-by-trial basis, without explicitly instructing subjects to integrate. This allowed us to establish that (a) subjects spontaneously integrate overlapping memories and (b) spontaneous integration during encoding is related to interference during retrieval. The fact that we specifically identified integration during encoding (i.e., as new associations were formed) is an important point given prior debates concerning the degree to which integration occurs during encoding vs. whether integration can instead be explained by associative dynamics that occur during retrieval^17,18,32–34^.

A primary focus of the present study was to tease apart measures of integration and reactivation as predictors of memory interference. To index memory reactivation, we used within-subject pattern classifiers (entirely independent from the integration classifier). We observed a positive relationship between trial-level measures of reactivation and integration, replicating our prior findings^20^ and consistent with the idea that memory integration requires reactivation. Despite this positive relationship between reactivation and integration, reactivation on its own did not predict interference costs at retrieval. At first pass, this result appears inconsistent with prior evidence associating reactivation during encoding with reduced interference-related forgetting^8,9^ or even with the results from Experiments 3 and 4, which clearly demonstrate that (re)activation of overlapping memories during encoding dramatically reduced interference during retrieval. However, the key idea motivating the current study is that the phenomenon of reactivation is dissociable from the mechanism of integration. Put another way, reactivation, on its own, does not guarantee a particular consequence. For example, in some contexts reactivation during encoding may reflect a shift of the memory system toward a retrieval state and away from a state that supports encoding new information^20,35^. Or, in the extreme, reactivation may even lead to differentiation of overlapping memories^22,23^. Although differentiation is computationally distinct from integration, it can also lead to reduced memory interference^36^ and may even co-occur with integration^37^. While the current findings do not directly address whether or not differentiation co-occurred with integration—or whether differentiation also contributed to reduced interference—it is notable that our findings highlight a unique benefit of integration. Namely, to the extent that overlapping memories are sufficiently well integrated, then activating a “non-target” memory can potentially facilitate retrieval of an overlapping, target memory. This is precisely what we observed in Experiments 3 and 4 where accuracy was significantly higher in the Old condition than the Novel condition (qualitatively reversing the interference effect seen in Experiments 1 and 2). A similar trend was observed in the fMRI experiment, with marginally faster RTs in the Old than Novel condition when integration evidence was high during AC learning. Thus, the current findings strongly underscore the importance of teasing apart the phenomenon of reactivation from the computations that are performed upon—or triggered by—reactivated memories^20,38^.

To the extent that reactivation can have distinct consequences, this raises an important question: What factors determine these consequences? Two factors that are likely relevant—and interrelated—are the amount of feature overlap between memories^39,40^ and the strength with which overlapping memories are reactivated^21,23^. For example, it is almost certain that Experiments 3 and 4, by virtue of their design, evoked stronger (re)activation of overlapping memories than did the fMRI study and this potentially influenced the strength and/or probability of integration. Other factors that may determine the consequences of reactivation include the temporal structure and sequencing of learning^13,38,41,42^ and task demands or goals^14,36,43^. While full consideration of this issue is beyond the scope of the present study, our methods, approach, and findings highlight the importance of—and a means for—characterizing the specific mechanisms at encoding that reduce interference during retrieval.

## Methods

### Experiments 1 and 2: Participants

A total of 40 paid subjects were included in Experiment 1 and a separate set of 40 paid subjects was included in Experiment 2. Subjects were recruited from the New York University community. Subjects in Experiment 1 were between the ages of 18 and 33 years (mean age = 21.8; 31 female). Subjects in Experiment 2 were between the ages of 19 and 31 (mean age = 22.3; 32 female). An additional four subjects participated in Experiment 1, but were excluded from analysis for either not following experimental instructions (*n* *=* 1) or for correctly responding to <10% of all AC test trials (*n* *=* 3). All subjects had normal or corrected-to-normal vision. Informed consent was obtained from all subjects and the study protocol was approved by the New York University Committee on Activities Involving Human Subjects.

### Experiments 1 and 2: Materials

Stimuli consisted of 126 words and 294 colored images. Words were a mix of verbs, adjectives, and nouns with a length between 3 and 11 letters (*M* = 6.0) and with SUBTLEX log frequency scores (http://subtlexus.lexique.org/) ranging from 1.3 to 4.2 (*M* *=* 2.9). The images consisted of colored photographs from three visual categories: famous people (e.g., Barack Obama; faces), famous locations (e.g., Times Square; scenes), and common objects (e.g., guitar; objects). For each subject, all words (A) were first randomly assigned to one of the three conditions (Old, Novel, Scrambled). Then, each word was randomly assigned two images (B and C) with the constraints that: (1) the two images were from different visual categories and (2) within each condition (Old, Novel, Scrambled), there were exactly seven B/C image pairs from each of the six possible groups (i.e., B = face/C = scene, B = face/C = object, B = scene/C = face, etc.). The remaining set of 42 images (14 from each visual category) was used for the distractor images in the Novel condition (see below). For the Scrambled condition, the distractor images were created using a custom MATLAB script (https://www.st-andrews.ac.uk/~jma23/code/phaseScrambleImage.m) that replaced the phase spectrum of each B image with uniform noise, while keeping the image’s Fourier power spectrum constant.

### Experiments 1 and 2: Procedures

The experiment consisted of three parts: AB Study and Test, AC Study and Test, and a Post Test. Note: Experiments 1–4 each included a Post Test, but the details and results of this post test are not included here.

Participants initially learned to associate words (A) with images of faces, scenes, or objects (B). During an AB Study trial, a word was presented directly above an image for 4 s. In Experiment 1, the image names (e.g., “Times Square”) were presented below the image during Study trials to facilitate subsequent verbal recall during Test trials. A white fixation cross was presented for 1 s in between trials. AB Study trials were grouped into 7 blocks of 18 trials. After each study block, participants were tested on their memory for the AB associations studied within the immediately preceding block. During an AB Test trial, a studied word (A) was presented in an empty box centered on the screen for 500 ms. The word then disappeared and the empty box remained on screen for an additional 3500 ms. Participants were instructed to recall the B image that had been paired with the word. The mode of response varied across the two experiments. In Experiment 1, subjects responded aloud by verbally naming the retrieved B image. Responses were transcribed, by hand, by an experimenter. In Experiment 2, subjects indicated the category (face/scene/object/not sure) of the B image using the keyboard. A fixation cross was presented for 1 s in between trials. Subjects’ responses were recorded from the onset of the cue until the end of the 1 s inter-trial fixation cross. For both experiments, the order of AB Study trials and the order of AB Test trials within each block was randomized. Additionally, each Study and Test block contained an equal number of trials from each condition (Old, Novel, Scrambled), an equal number of B images from each visual category (face, object, scene), and an equal number of B images from each visual category within each condition (i.e., 2 images × 3 visual categories × 3 conditions).

After studying all of the AB associations, subjects studied each word (A) paired with a new image (C). During an AC Study trial, a previously studied word (A) was presented directly above a new image (C). Subjects were told that each previously studied word would be paired with a new image and that their task was to learn the new word-image pairing. Identical to the AB Study trials, each pair was presented for 4 s with a 1 s fixation cross in between trials. As with the AB Study trials, the AC Study trials were grouped into 7 blocks of 18 trials each, with each AC Study block followed by an AC Test block that tested memory for the AC associations studied within the immediately preceding block. AC Test trials were identical to AB Test trials except for one critical difference. All AC Test trials were preceded by a briefly presented (200 ms) distractor image. There were three distractor conditions: Old, Novel, and Scrambled. In the Old condition, the distractor image was the original B image paired with the cued word during the AB Study trials. In the Novel condition, the distractor image was a previously unseen (novel) image from the same visual category as the corresponding B image. In the Scrambled condition, the distractor image was a phase-scrambled version of the B image (see Methods). Subjects were told that the distractor images were irrelevant to their task, but subjects were instructed not to look away or blink when the distractor images appeared. Note: the test trials were also specifically designed to discourage subjects from looking away from the distractor image and/or closing their eyes. Namely, the cue words were presented in the center of the screen in the same location as the distractor images and the words only appeared for a brief period of time (500 ms) immediately following the distractor image. For each Experiment, the response method during the AC Test trials matched the response method for the AB Test trials (i.e., Experiment 1 = verbal response, Experiment 2 = button-press response).

Each AC Study and Test block contained an equal number of trials from each condition (Old, Novel, Scrambled), an equal number of C images from each visual category (face, object, scene), and an equal number of C images from each visual category within each condition (i.e., 2 images × 3 visual categories × 3 conditions). To reduce variance in temporal lag between corresponding AB and AC trials, the assignment of words to block number was consistent across the AB and AC phases—that is, words studied and tested in AB block 1 were then studied and tested in AC block 1, and so on. However, the order of AC Study trials and the order of AC Test trials within each block was randomized. Furthermore, each AC Study and Test block contained one trial from each of the 6 possible B/C image category pairings (e.g., B = face, C = object) per condition.

Given the inevitable variability that arose in subject’s verbal responses during test, we sought to create an objective scoring scheme that could be applied across subjects and experiments. In our scoring scheme, a subject’s response was counted as correct if the subject’s description characterized <10% of images in the target image’s category (face, scene, or object). For example, if subjects responded “Paris” instead of responding with the given image label “Arc de Triomphe,” their response would be coded as correct since <10% of scene images in the experiment were landmarks in Paris. However, had they responded “building” their response would be coded as incorrect since more than 10% of images could be characterized as buildings.

### Experiments 3 and 4: Participants

A total of 40 paid subjects were included in Experiment 3 and a separate set of 40 paid subjects was included in Experiment 4. Subjects were recruited from the New York University community. In Experiment 3, all subjects were between the ages of 18 and 33 (mean age = 22.8; 33 female) and had normal or corrected-to-normal vision. An additional four subjects participated in Experiment 3, but were excluded from the analysis for either not following experimental instructions (*n* *=* 1) or for correctly responding to <10% of all AC test trials (*n* *=* 3). In Experiment 4, all participants were between the ages of 18 and 35 (mean age = 21.4; 26 female) and had normal or corrected-to-normal vision. An additional three subjects participated in Experiment 4, but were excluded from analysis for technical errors (*n* = 2) or for correctly responding to <10% of all AC test trials (*n* = 1). Informed consent was obtained from all subjects and the study protocol was approved by the New York University Committee on Activities Involving Human Subjects.

### Experiments 3 and 4: Materials

Materials were identical to Experiments 1 and 2.

### Experiments 3 and 4: Procedures

The procedures for Experiments 3 and 4 were identical to Experiment 1 with one critical change. Immediately prior to all AC Study trials, subjects were reminded of the original B association. This reminder differed across experiments. Broadly: in Experiment 3, each AC Study trial was preceded by a cue to recall the original (and corresponding) B image, whereas in Experiment 4, the B image was briefly presented just prior to each AC Study trial. In Experiment 3, each AC Study trial began with the presentation of a word cue (A) presented above a blank box and subjects had 4000 ms to retrieve and verbally name the corresponding B image (similar to a test trial). Following a 1000 ms fixation cross, the same word cue was presented above a new (C) image for 4000 ms and subjects were instructed to study this new association (AC) in preparation for a subsequent test. In Experiment 4, immediately prior to each AC Study trial, the B image was presented on screen for 200 ms. Following the presentation of the B image, a word cue (A) was presented with a new (C) image for 4000 ms. In Experiment 4, subjects were told that the briefly presented B image was irrelevant to their task (learning the AC pairs). For both Experiments 3 and 4, there were no explicit instructions to integrate across the B and C images.

### Behavioral pilot for fMRI experiment: Participants

A total of 21 paid subjects were included in the behavioral pilot for the fMRI study. Subjects were recruited from the New York University community. All subjects were between the ages of 18–31 (mean age = 22.7; 16 female) and had normal or corrected-to-normal vision. Informed consent was obtained from all subjects and the study protocol was approved by the New York University Committee on Activities Involving Human Subjects.

### Behavioral pilot for fMRI experiment: Materials

In the behavioral pilot for the fMRI experiment, the Scrambled condition was no longer included (see Procedure). Therefore, the number of stimuli used was slightly different from Experiments 1–4. A total of six words were removed from the set used in Experiments 1–4 and six images were added (three in each visual category), resulting in a stimulus set comprised of 120 words and 300 images. For each subject, 240 of the images were assigned to words (two images from different visual categories were assigned to each word). The remaining 60 images (20 from each visual category) were used as novel distractor images during the Test phase. As in the previous experiments, the image assignments were controlled so that within each condition (Old, Novel) there were exactly 10 B/C image pairs from each of the six possible groups (i.e., B = face/C = scene, B = face/C = object, B = scene/C = face, etc.).

### Behavioral pilot for fMRI experiment: Procedures

The task paradigm in the behavioral pilot for the fMRI experiment was most similar to the paradigm used in Experiment 2. However, in order to focus on the critical comparison between Old and Novel trials, the Scrambled condition was no longer included. Additionally, we added an AB Exposure phase that was intended to strengthen the AB associations, and, thereby, increase the probability that B images would be spontaneously reactivated during AC encoding.

During the AB Exposure phase, subjects were exposed to all 120 AB associations. During each trial, a word (A) was presented above an image (B) for 3 s. During that time, participants were instructed to subjectively rate how well the word and the image were paired together on a four-point scale (poor, fair, good, great) using the keyboard. A fixation cross was presented for 1 s in between trials. The rating task was only intended to encourage elaborative encoding of the AB associations.

After the AB Exposure phase, subjects completed alternating AB Study and Test blocks. The timing and structure of the AB Study and Test trials were identical to Experiment 2. Here, however, subjects completed 10 Study/Test cycles and each block within each cycle contained 12 trials (six per condition) with exactly two faces, two scenes, and two objects studied and tested per condition.

After all of the AB Study/Test cycles were completed, subjects completed AC Study/Test cycles, as in the prior experiments. There were a total of 10 AC Study/Test cycles. The timing and structure of the AC Study and Test trials were identical to Experiment 2.

### fMRI experiment: Participants

A total of 20 paid subjects participated in the fMRI experiment. All participants were between the ages of 19 and 33 (mean age = 24.2 years; 17 females), right-handed, and had normal or normal-to-corrected vision. An additional two subjects participated, but were excluded from analysis due to technical issues with the scanner. Informed consent was obtained from all subjects and the study protocol was approved by the New York University Committee on Activities Involving Human Subjects.

### fMRI experiment: Materials

The stimuli for the fMRI experiment included all of the same materials as the behavioral pilot, plus an additional 42 colored images (14 per visual category) that were specifically added for use in the Visual Category Localizer (described below).

### fMRI experiment: Procedures

The experiment consisted of four parts: AB Exposure, AB Study/Test, AC Study/Test, and Visual Category Localizer. All AB learning (Exposure, Study, and Test trials) occurred before subjects entered the scanner. All AC learning (Study and Test trials) and the Visual Category Localizer was completed during fMRI scanning.

The procedures for the AB Exposure, AB Study, and AB Test were identical to the behavioral pilot.

Each AC Study/Test cycle was completed during a separate fMRI run; thus, the 10 AC Study/Test cycles corresponded to 10 fMRI runs. Each cycle started with a message reading “Get ready: Study Round [number]!,” which was displayed for 6 s. This message was followed by a 4-s fixation cross. The Get Ready and fixation cross screens (10 s total) allowed for T1 equilibration. After the fixation cross, the first AC Study trial began. The trial timing was identical to the behavioral pilot, except that, here, the inter-trial interval (ITI) contained a series of numbers and subjects indicated, via an MRI compatible button box, whether each number was odd or even. Specifically, each ITI (6 s) began with the presentation of a fixation cross (1.5 s), followed by two numbers (1 s each) with a fixation cross (1 s) in between each number. The last number was followed by a fixation cross (1.5 s) before the start of the next trial. This “active baseline” task was used during the ITI in order to reduce covert rehearsal of the associations (which would not be captured by our fMRI analyses). After each AC Study block was completed, a message reading “Get ready: Test Round [number]!” appeared for 6 s, followed by AC Test trials. The AC Test trials were identical to the behavioral pilot except that (a) the ITI was longer (6 s) and was identical to the “active baseline” used during the AC Study trials and (b) subjects indicated the category of the C image (face/scene/object/not sure) using a button box.

Following the 10 AC Study/Test cycles, participants completed two runs of a visual category localizer scan. Each localizer run contained images from three visual categories: faces, scenes, and objects. Each trial (2 s) consisted of a “mini-block” of four images from the same visual category, presented in rapid succession. Each image was presented briefly (400 ms) with a blank screen (100 ms) in between images. Each trial was followed by a 6-s fixation cross. The “mini-block” structure was modeled after a prior study^44^ and was intended to boost efficiency in detecting category-specific signals. Each run contained 45 trials (15 trials per condition). Participants pressed a button whenever they detected that an image repeated within a trial (i.e., within a four-image mini-block), which occurred on 9 out of 45 trials in a run (six trials per visual category). Each run started with a message reading “Get ready: Repetition Round [number]!” for 6 s, followed by a 4-s fixation cross. The images used in the localizer scan consisted of the 300 images from the main experiment plus an additional 42 images (14 from each visual category). The set of 342 images was divided evenly across two localizer scans with the 42 new images randomly interspersed with the 300 images from the main experiment.

### fMRI acquisition

fMRI scanning was performed on the 3 T Siemens Allegra head-only scanner at the Center for Brain Imaging at New York University using a Siemens head coil. Structural images were collected using a T1-weighted magnetization-prepared rapid acquisition gradient echo anatomical volume (256 × 256 matrix, 176 1-mm sagittal slices, 1 × 1 × 1 mm voxels). Functional images were acquired parallel to the anterior commissure–posterior commissure axis using a single-shot EPI sequence (repetition time = 2 s; echo time = 30 ms; field of view = 192 × 240 mm, flip angle = 82°, bandwidth = 4165 Hz/px, and echo spacing = 0.31 ms). For all functional scanning, we obtained 35 contiguous oblique–axial slices (3 × 3 × 3-mm voxels) per volume.

There were a total of 10 AC Study/Test cycles, with each cycle corresponding to an fMRI scan that consisted of 128 volumes (4 m 16 s). Of the 128 volumes, the first five were discarded to account for T1 stabilization (during this time, subjects saw a “Get ready: Study Round [number]!” screen and then a fixation cross). The next 60 volumes corresponded to the AC Study trials, followed by three volumes during which subjects had a momentary break and a reminder of the upcoming Test trials (“Get ready: Test Round [number]!”). The final 60 volumes corresponded to the AC Test trials. The visual category localizer was collected across two fMRI scans. Each scan consisted of 185 volumes (6 m 10 s). The first five volumes were discarded to account for T1 stabilization (during this time subjects saw a “Get ready: Repetition Round [number]!” screen followed by a fixation cross).

### fMRI pre-processing

Images were preprocessed using FSL (FMRIB’s Software Library, Oxford, UK). First, each time series was realigned to the middle volume within each run to correct for head motion. All functional images were spatially smoothed using an 8-mm full-width at half-maximum gaussian kernel to facilitate across-subject decoding analyses. Images were high-pass filtered with a 128-s filter. The images from each participant were then normalized to Montreal Neurological Institute (MNI) standard space using ANTs (Advance Normalization Tools; picsl.upenn.edu/software/ants/) version 2.1.0 (ANTsIntroduction.sh script to MNI 152 2 mm template). First, ANTs was used to compute the coregistration parameters from each participant’s functional space to their high-resolution T1-weighted anatomical scan using rigid affine transformation. Then, each participant’s anatomical scan was normalized to FSL’s MNI 152 template using a symmetric diffeomorphic transformation. Those transformation parameters were then applied to each functional time series to normalize them to the common template.

### Mnemonic state decoding

We hypothesized that the level of interference between the B and C images during retrieval would, in part, be determined by the “state” of the memory system during AC encoding. Specifically, we hypothesized that greater integration during AC Study trials would predict less interference (in the Old condition) during AC Test trials. To assess the state of the memory system during AC Study trials, we utilized data from an independent, previously described fMRI experiment^20^ to train a pattern classifier to discriminate between different memory states. In the prior study, subjects studied overlapping word-image associations, similar to the current fMRI experiment (but without any distractor images during). Instead, prior to each AC Study trial, subjects received one of three instructions: Retrieve, Encode, or Integrate. The Retrieve instruction signaled that, for the upcoming AC association, subjects should retrieve the original AB association (and ignore the new C image). The Encode instruction signaled that subjects should focus on encoding the new AC association. The Integrate instruction signaled that subjects should update the prior association (AB) to include the new association (AC).

In our prior study^20^, we trained a pattern classifier to discriminate between the three mnemonic states (retrieve, encode, integrate) using whole-brain fMRI activity patterns and leave-one-subject-out cross-validation. The prior study established three key points: (1) we were able to decode processing states, including discriminating an integration state from encoding or retrieval states, (2) classifier evidence for an integration state predicted performance on a subsequent test of subjects’ ability to link the AB and AC associations, and (3) when the trained classifier was applied to an independent set of subjects that were not given any explicit instructions on how to process the overlapping associates, classifier evidence for an integration state during AC encoding again predicted subjects’ ability to later link across the AB and AC associations. Thus, we have convincingly established that memory states can be decoded from fMRI activity patterns using across-subject pattern classification, and, critically, that these decoded memory states are behaviorally relevant. The classifier approach and data from Richter et al.^20^ have also been successfully applied to another, independent data set to predict memory outcomes^38^.

To optimize the application of the data from Richter et al.^20^ to the current fMRI study, we re-processed the raw data from the original Richter et al.^20^ experiment using the same pre-processing pipeline as the current fMRI experiment (including the use of ANTs for normalization). As in the Richter et al.^20^ study, the images were then de-trended and *z*-scored within run and all classification analyses were run on the “raw” (un-modeled) data. Whole-brain activity patterns were used for mnemonic state decoding because the whole-brain ROI outperformed all individual ROIs in the Richter et al.^20^ study. The specific volumes that were used for training the classifier were identical to the Richter et al.^20^ study. Specifically, volumes 4–6 (4–10 s post-trial onset) were averaged together to obtain a single spatial pattern per trial. Each of these trials was labeled according to the mnemonic state instruction the participant received on that trial (encode, retrieve, or integrate). Data from all 20 subjects in the Richter et al.^20^ study was used to train the classifier. However, before proceeding, we compared several different types of classification algorithms available in LIBLINEAR from Sci-kit Learn. In the original study by Richter et al.^20^, we used L2-regularized logistic regression, which was an a priori decision based on prior data sets^26,27^. However, the Richter et al.^20^ study differed from our prior studies in terms of the kind of information that was being decoded (i.e., mnemonic states) and it is possible that this factor is relevant to selecting an optimal classification algorithm (although it was not a factor we previously considered). Indeed, we found that re-analyzing the Richter et al.^20^ data using leave-one-subject-out support vector classification (SVC class with RBF kernel, penalty parameter = 1) yielded markedly higher decoding accuracy (*M* *=* 47.4%) of the mnemonic states (retrieve, encode, integrate) relative to the leave-one-subject-out L2-regularized logistic regression algorithm that we originally used (*M* *=* 39.4 %; difference in performance between classifiers: *t*_20_ = 4.73, *p* < 0.001). Thus, based on this substantial improvement in classification performance for data from the prior study, we opted to train a classifier on the Richter et al.^20^ data using the SVC and then apply this trained classifier to data from the current fMRI experiment.

To test the mnemonic state classifier (trained on data from Richter et al.^20^), we applied the classifier to every AC Study trial in the current fMRI experiment (averaging the three volumes corresponding to 4–10 s post-trial onset). For each trial, the classifier generated three values corresponding to the three mnemonic states: retrieval, encoding, and integration. To correct for the non-normality in the distribution of these raw classifier values, the raw values (*p*) were transformed into log-probability estimates [log(*p*)]. We refer to the log-transformed values as “classifier evidence.” Note: because of the log transformation, classifier evidence values were negative.

### Category reactivation decoding

To test for reactivation of B images during AC Study, we trained a classifier (L2-regularized logistic regression), using data from the Visual Category Localizer, to discriminate between the three visual categories (face, scene, object). Specifically, data were concatenated across the two localizer scans and the activity pattern corresponding to each trial was defined as the average of the two volumes corresponding to 4–8 s post-trial onset. The classifier was trained using a VTC region of interest, consistent with prior studies from our group^26^ and motivated by our prior finding that integration evidence positively correlates with reactivation in VTC^20^.

A VTC region of interest was constructed by combining the “temporal occipital fusiform” and “posterior fusiform” labels from the Harvard–Oxfrod atlas. The trained classifier was tested on each trial from the AC Study phase, averaging the three volumes corresponding to 4–10 s post-trial onset (as with the mnemonic state classifier). For each AC Study trial, one visual category corresponded to the new (C) image, one category corresponded to the Old (B) image, and the final category (neither B nor C) served as a baseline category. As a basic validation step, we first tested the accuracy with which the classifier “guessed” the correct visual category of the C image (i.e., the image that subjects actually saw on each trial). Mean classification accuracy was 84.17% (chance = 33.33%), clearly establishing that the classifier was successful in decoding visual category information. To measure reactivation, however, we used logit-transformed classifier values that we refer to as “classifier evidence.” As noted above, these values were negative due to the logit transformation. For each trial, reactivation strength was defined as the difference in classifier evidence for the category of the B image vs. the baseline category (i.e., B category—baseline category). Thus, if there was greater classifier evidence for the B category than the baseline category, this yielded a positive reactivation score (even though classifier evidence scores were negative).

### Statistical analyses

Behavioral and fMRI data were analyzed using a combination of repeated-measure ANOVAs, follow-up paired *t* tests, and mixed-effects models. Tests of normality were not included given that ANOVAs and *t* tests are generally robust to violations of normality—particularly with larger sample sizes. For all analyses we used a threshold of *p* < 0.05 to establish statistical significance. Corrections for multiple comparisons were not applied/applicable given that all critical statistical analyses involved a single test or comparison corresponding to a resulted that was predicted a priori. All *t* tests were two-tailed. All statistical analyses run on RTs (in Experiment 2, the behavioral pilot for the fMRI study, and the fMRI Experiment) were performed on the log transform of each RT value to correct for non-normality in the distribution. Mixed-effects models were implemented in the lme4 package for R (http://lme4.r-forge.r-project.org). The significance of predictors was assessed using likelihood ratio tests on nested models.

### Reporting summary

Further information on research design is available in the Nature Research Reporting Summary linked to this article.

## Supplementary information

Supplementary Information

Peer Review File

Reporting Summary

## Data Availability

Data are available upon request from the corresponding author.

## Code availability

Code to run the experiments and analyze the data are available upon request from the corresponding author.
