# Supplementary material for: Interference between overlapping memories is predicted by neural states during learning
Source: Nat Commun. 2019 Nov 25;10:5363. doi: 10.1038/s41467-019-13377-x (PMC6877550; doi:10.1038/s41467-019-13377-x)
Supplement: Supplementary file 1 — Supplementary Information [file 41467_2019_13377_MOESM1_ESM.pdf]

SUPPLEMENTARY TABLES

	Accuracy (%)	Reaction Time (ms)
**Experiment 1**	54.78 (19.96)	-----
**Experiment 2**	70.52(13.09)	1447.7 (336.9)
**Experiment 3**	61.33 (13.18)	-----
**Experiment 4**	52.06 (17.87)	-----
**fMRI Pilot**	84.44 (14.45)	1742.42 (195.6)
**fMRI Experiment**	91.58 (5.56)	1815.41 (291.5)

Supplementary Table 1. AB Test accuracy and reaction time data for each experiment. Each cell contains mean accuracy or reaction time (for correct trials only), with standard deviation in parentheses. Reaction times were only recorded for Experiment 2. Note: Experiments 1, 3 and 4 used verbal report to measure item-specific recall (e.g., “Barack Obama”) whereas Experiment 2 used button-presses to measure recall for the visual category of each item (e.g., “scene”).

	Old (%)	Novel (%)	Scrambled (%)
**Experiment 1**	44.40 (21.73)	47.32 (21.5)	50.62 (21.09)
**Experiment 2**	67.50 (21.34)	69.35 (19.67)	69.70 (18.34)
**Experiment 3**	33.15 (14.28)	30.65 (13.00)	31.85 (13.60)
**Experiment 4**	43.15 (18.14)	39.20 (19.54)	43.15 (19.46)
**fMRI Pilot**	70.34 (16.24)	72.62 (16.22)	-----
**fMRI Experiment**	80.50 (12.66)	82.75 (11.15)	-----

Supplementary Table 2. AC Test accuracy data for each experiment. Mean accuracy for each condition (column) and experiment (row), with standard deviation in parentheses. Notes: the Scrambled condition was not included in the fMRI Pilot or fMRI Study; Experiments 1, 3 and 4 used verbal report to measure item-specific recall (e.g., “Barak Obama”) whereas Experiment 2, the fMRI Pilot, and the fMRI Study used button-presses to measures memory for the visual category of each item (e.g., “Scene”).

	Old (ms)	Novel (ms)	Scrambled (ms)
**Experiment 1**	-----	-----	-----
**Experiment 2**	2064.69 (392.3)	1991.37(429.1)	1860.58 (340.7)
**Experiment 3**	-----	-----	-----
**Experiment 4**	-----	-----	-----
**fMRI Pilot**	2037.70 (234.6)	1917.88 (277.8)	-----
**fMRI Experiment**	2277.24 (357.6)	2283.15 (366.0)	-----

Supplementary Table 3. **AC Test reaction time data for each experiment.** Mean reaction times (correct trials only) for each condition (column) and experiment (row), with standard deviation in parentheses. Notes: the Scrambled condition was not included in the fMRI Pilot or fMRI Study; reaction times were not recorded for Experiments 1, 3, and 4 (which relied on verbal recall).
