# Supplementary material for: Interference between overlapping memories is predicted by neural states during learning
Source: Nat Commun. 2019 Nov 25;10:5363. doi: 10.1038/s41467-019-13377-x (PMC6877550; doi:10.1038/s41467-019-13377-x)
Supplement: Supplementary file 3 — Reporting Summary [file 41467_2019_13377_MOESM3_ESM.pdf]

nature research

Corresponding author(s): Brice Kuhl

Last updated by author(s): May 30, 2019

Reporting Summary

Nature Research wishes to improve the reproducibility of the work that we publish. This form provides structure for consistency and transparency in reporting. For further information on Nature Research policies, see Authors & Referees and the Editorial Policy Checklist.

Statistics

For all statistical analyses, confirm that the following items are present in the figure legend, table legend, main text, or Methods section.

n/a	Confirmed	
☐	☒	The exact sample size (*n*) for each experimental group/condition, given as a discrete number and unit of measurement
☐	☒	A statement on whether measurements were taken from distinct samples or whether the same sample was measured repeatedly
☐	☒	The statistical test(s) used AND whether they are one- or two-sided<br>*Only common tests should be described solely by name; describe more complex techniques in the Methods section.*
☒	☐	A description of all covariates tested
☐	☒	A description of any assumptions or corrections, such as tests of normality and adjustment for multiple comparisons
☐	☒	A full description of the statistical parameters including central tendency (e.g. means) or other basic estimates (e.g. regression coefficient) AND variation (e.g. standard deviation) or associated estimates of uncertainty (e.g. confidence intervals)
☐	☒	For null hypothesis testing, the test statistic (e.g. *F*, *t*, *r*) with confidence intervals, effect sizes, degrees of freedom and *P* value noted<br>*Give P values as exact values whenever suitable.*
☒	☐	For Bayesian analysis, information on the choice of priors and Markov chain Monte Carlo settings
☒	☐	For hierarchical and complex designs, identification of the appropriate level for tests and full reporting of outcomes
☒	☐	Estimates of effect sizes (e.g. Cohen's *d*, Pearson's *r*), indicating how they were calculated

Our web collection on statistics for biologists contains articles on many of the points above.

Software and code

Policy information about availability of computer code

Data collection	Data were collected in MATLAB 7.11 using PsychToolbox 3.0.11
Data analysis	Preprocessing for the fMRI data was run with FSL 5.0.9 and ANTs 1.9.1 using the python package lyman 0.0.6. Classification analyses on the fMRI data were run using the sci-kit learn python package. Statistics for all experiments were computed using R.

For manuscripts utilizing custom algorithms or software that are central to the research but not yet described in published literature, software must be made available to editors/reviewers. We strongly encourage code deposition in a community repository (e.g. GitHub). See the Nature Research guidelines for submitting code & software for further information.

Data

Policy information about availability of data

All manuscripts must include a data availability statement. This statement should provide the following information, where applicable:

- Accession codes, unique identifiers, or web links for publicly available datasets
- A list of figures that have associated raw data
- A description of any restrictions on data availability

Data from the experiment is available on request form the corresponding author.

Field-specific reporting

Please select the one below that is the best fit for your research. If you are not sure, read the appropriate sections before making your selection.

☐ Life sciences ☒ Behavioural & social sciences ☐ Ecological, evolutionary & environmental sciences

Behavioural & social sciences study design

All studies must disclose on these points even when the disclosure is negative.

Study description	The data are quantitative experimental data
Research sample	Subjects were all recruited form the New York Univesrsity community. In total, 201 people participated in the experiments (155 females; mean 22.3 years of age).
Sampling strategy	The target sample size for the behavioral studies was set to 40 based on norms for similar studies in our field. We did not have an a priori estimate of effect size that we could use to determine the sample size. However, we also included multiple behavioral studies so that we could test for consistency of critical behavioral effects across independent studies. The target sample size for the fMRI study was set to 20 subjects based on a similar experiment from our lab (Ricther, Chanales & Kuhl 2016).
Data collection	Behavioral experiments were collected on Mac computers running Matlab 7.11. Participants were seated in a testing room with an experimenter who was aware of the study hypothesis. In experiments 1, 3, 4, and behavioral pilot the experimenter in the room recorded the verbal responses of the participant using a pen and paper. In experiment 2 participants responded using a keyboard. The fMRI data were collected using Siemens Allegra head-only scanner and their responses were recorded using a button box while they were in the scanner.
Timing	Data collection started on April 17th 2013 and terminated on July 24th 2015.
Data exclusions	Exclusion criterion were pre-established and applied identically across all experiments. Participants were excluded for either not following experimental instructions, poor memory performance (correctly responding to less than 10% of AC test trials, or for technical errors.<br>Exp 1: not following experimental instructions (n = 1); correctly responding to less than 10% of all AC test trials (n = 3)<br>Exp 2: no exclusions<br>Exp 3: not following experimental instructions (n = 1); correctly responding to less than 10% of all AC test trials (n = 3)<br>Exp 4: technical error (n =2); correctly responding to less than 10% of all AC test trials (n = 3)<br>fMRI Behavioral Pilot: no exclusions<br>fMRI: technical issues (n =2)
Non-participation	No participants dropped out or declined participation.
Randomization	Participants were allocated to experiments based on when they signed up for the study.

Reporting for specific materials, systems and methods

We require information from authors about some types of materials, experimental systems and methods used in many studies. Here, indicate whether each material, system or method listed is relevant to your study. If you are not sure if a list item applies to your research, read the appropriate section before selecting a response.

Materials & experimental systems

n/a	Involved in the study	
☒	☐	Antibodies
☒	☐	Eukaryotic cell lines
☒	☐	Palaeontology
☒	☐	Animals and other organisms
☐	☒	Human research participants
☒	☐	Clinical data

Methods

n/a	Involved in the study	
☒	☐	ChIP-seq
☒	☐	Flow cytometry
☐	☒	MRI-based neuroimaging

Human research participants

Policy information about studies involving human research participants

Population characteristics	See above.
Recruitment	Participants were recruited from the NYU community through flyers posted online and on campus.
Ethics oversight	New York University Committee on Activities Involving Human Subjects

Note that full information on the approval of the study protocol must also be provided in the manuscript.

Magnetic resonance imaging

Experimental design

Design type	The study was an event-related design
Design specifications	Each subject completed 10 runs of AC Study and Test and 2 runs of the visual localizer. Each AC Study and Test run contained 12 study trials and 12 test trials. Each study and test trial was 4s with a 6s ITI. Each visual localizer run contained 45 trials. Each trial was 4s with a 6s ITI.
Behavioral performance measures	Correct button responses and RT were recorded for AC Test trials and the visual localizer runs.

Acquisition

Imaging type(s)	functional and structural
Field strength	3 Tesla
Sequence & imaging parameters	Structural images were collected using a T1- weighted magnetization-prepared rapid acquisition gradient echo anatomical volume (256 × 256 matrix, 176 1-mm sagittal slices, 1 x 1 x 1 mm voxels). Functional images were acquired parallel to the anterior commissure– posterior commissure axis using a single-shot EPI sequence (repetition time = 2 s; echo time = 30ms; field of view = 192 × 240 mm, flip angle = 82°, bandwidth = 4165 Hz/px and echo spacing = 0.31 ms). For all functional scanning, we obtained 35 contiguous oblique-axial slices (3 × 3 × 3-mm voxels) per volume.
Area of acquisition	whole brain

Diffusion MRI ☐ Used ☒ Not used

Preprocessing

Preprocessing software	Images were preprocessed using FSL 5.0.9 (FMRIB's Software Library, Oxford,United Kingdom). First, each timeseries was realigned to the middle volume within each run to correct for head motion. All functional images were spatially smoothed using an 8 mm full-width at half maximum gaussian kernel to facilitate across-subject decoding analyses. Images were high-pass filtered with a 128-second filter.
Normalization	The images from each participant were normalized to Montreal Neurological Institute (MNI) standard space using ANTs (Advance Normalization Tools). First, ANTs was used to compute the coregistration parameters from each participant's functional space to their high-resolution T1-weighted anatomical scan using rigid affine transformation. Then, each participant's anatomical scan was normalized to FSL's MNI 152 template using a symmetric diffeomorphic transformation. Those transformation parameters were then applied to each functional timeseries to normalize them to the common template.
Normalization template	MNI 152 template.
Noise and artifact removal	Motion correction was performed during preprocessing.
Volume censoring	No volume censoring was performed.

Statistical modeling & inference

Model type and settings	Classification analyses were perfrormed on the 'raw' unmodeled data.
Effect(s) tested	No effects were tested

Specify type of analysis: ☐ Whole brain ☐ ROI-based ☐ Both

Statistic type for inference (See Eklund et al. 2016)	Not applicable
Correction	Not applicable

Models & analysis

n/a	Involved in the study
☒	☐ Functional and/or effective connectivity
☒	☐ Graph analysis
☐	☒ Multivariate modeling or predictive analysis

Multivariate modeling and predictive analysis	Mnemonic State Decoding: In our prior study (Richter, Chanales & Kuhl, 2016), we trained a pattern classifier to discriminate between the three mnemonic states (retrieve, encode, integrate) using whole-

brain fMRI activity patterns and leave-one-subject-out cross validation. Here, we trained a classifier (SVC class with RBF kernel) to decode the mneumonic states from this dataset. We then applied this classifier to every AC Study trial in the current fMRI experiment (averaging the three volumes corresponding to 4-10 s post-trial onset). For each trial, the classifier generated three values corresponding to the three mnemonic states: retrieval, encoding, and integration. To correct for the non-normality in the distribution of these raw classifier values, the values were logit-transformed.

Category Reactivation Decoding: To test for reactivation of B images during AC Study, we trained a classifier (L2-regularized logistic regression), using data from the Visual Category Localizer, to discriminate between the three visual categories (face, scene, object). Specifically, data was concatenated across the two localizer scans and the activity pattern corresponding to each trial was defined as the average of the two volumes corresponding to 4-8 s post-trial onset extracted from ventral temporal cortex. The trained classifier was tested on each trial from the AC Study phase, averaging the three volumes corresponding to 4-10 s post-trial onset (as with the mnemonic state classifier). Again, the classifier values were logit-transformed to correct for non-normality.
