# Supplementary material for: Interference between overlapping memories is predicted by neural states during learning
Source: Nat Commun. 2019 Nov 25;10:5363. doi: 10.1038/s41467-019-13377-x (PMC6877550; doi:10.1038/s41467-019-13377-x)
Supplement: Supplementary file 2 — Peer Review File [file 41467_2019_13377_MOESM2_ESM.pdf]

Editorial Note: This manuscript has been previously reviewed at another journal that is not operating a transparent peer review scheme. The manuscript was considered suitable for publication without further review at Nature Communications.
